# Clinical relevance of joint line obliquity after high tibial osteotomy for medial knee osteoarthritis remains controversial: a systematic review

**DOI:** 10.1007/s00167-023-07486-w

**Published:** 2023-06-20

**Authors:** Tianshun Xie, Reinoud W. Brouwer, Inge van den Akker-Scheek, Hugo C. van der Veen

**Affiliations:** 1 Department of Orthopaedic Surgery, University of Groningen, University Medical Center Groningen, P.O. Box 30.001, 9700 RB Groningen, The Netherlands; 2 Department of Orthopaedic Surgery, Martini Hospital, Groningen, The Netherlands

**Keywords:** Joint line obliquity, Patient-reported outcome, Cartilage, Ligament, Surgical survival, Upper limit, Medial knee osteoarthritis, High tibial osteotomy

## Abstract

**Purpose:**

To systematically review the literature on the association between knee joint line obliquity (KJLO) and clinical outcome after high tibial osteotomy (HTO) for medial knee osteoarthritis and summarize the KJLO cut-off value used when studying this association.

**Methods:**

A systematic search was conducted in three databases** (**PubMed, Embase, and Web of Science) on September 2022, updated on February 2023. Eligible studies describing postoperative KJLO in relation to clinical outcome after HTO for medial knee osteoarthritis were included. Nonpatient studies and conference abstracts without full-text were excluded. Two independent reviewers assessed title, abstract and full-text based on the inclusion and exclusion criteria. The modified Downs and Black checklist was used to assess the methodological quality of each included study.

**Results:**

Of the seventeen studies included, three had good methodological quality, thirteen fair quality, and one had poor quality. Conflicting findings were shown on the associations between postoperative KJLO and patient-reported outcome, medial knee cartilage regeneration, and 10-year surgical survival in sixteen studies. Three good-quality studies found no significant differences in lateral knee cartilage degeneration between postoperative medial proximal tibial angle > 95° and < 95°. Joint line orientation angles by the tibial plateau of 4° and 6°, joint line orientation angle by the middle knee joint space of 5°, medial proximal tibial angles of 95° and 98°, and Mikulicz joint line angle of 94° were KJLO cut-off values used in the included studies.

**Conclusion:**

Based on current evidence, the actual association between postoperative KJLO and clinical consequences after HTO for medial knee osteoarthritis cannot be ascertained. The clinical relevance of KJLO after HTO remains controversial.

**Level of evidence:**

IV.

## Introduction

**Fig. 1:**
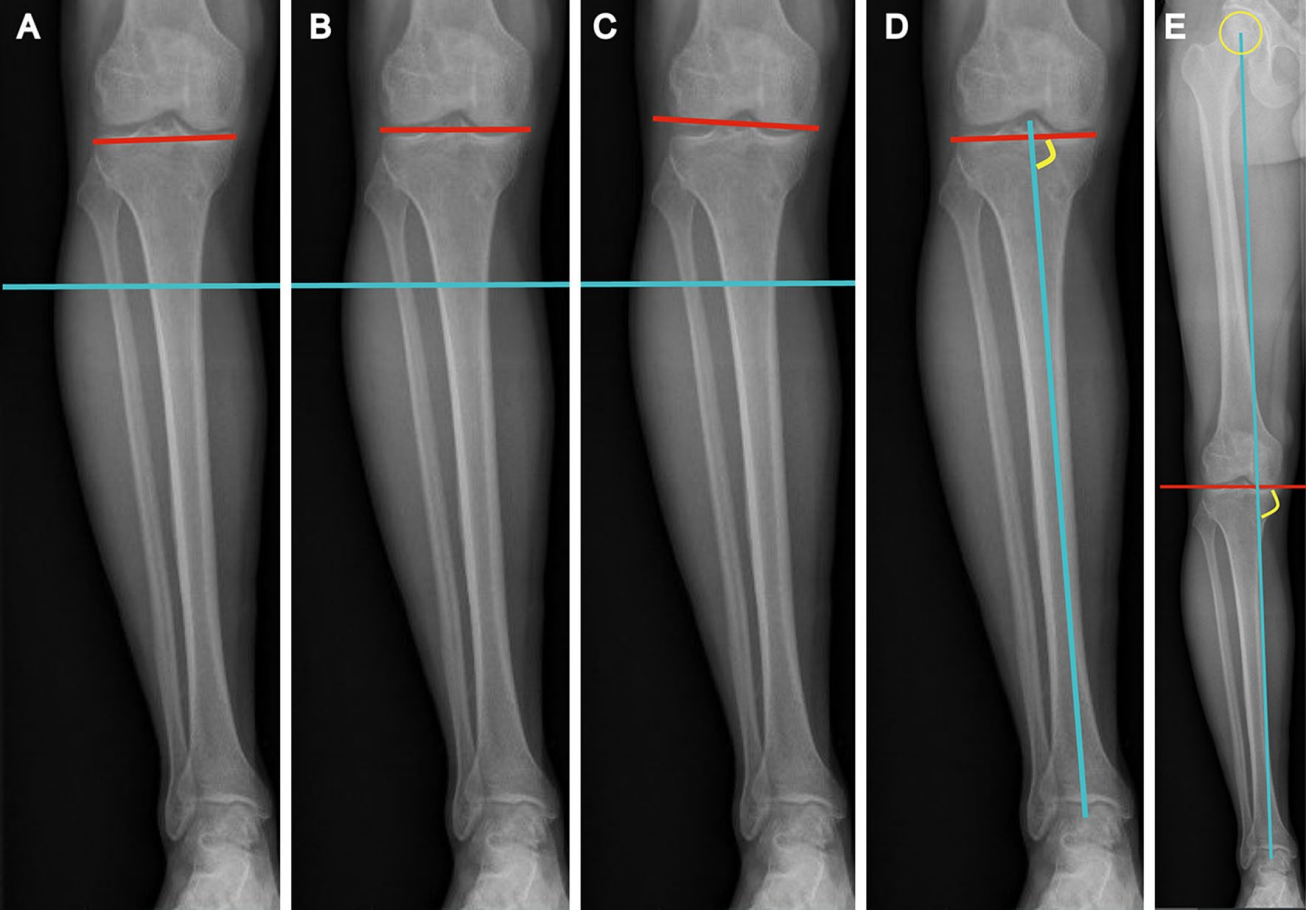
KJLO measurement methods illustrated on anteroposterior long-standing radiograph. **A** Joint line orientation angle by the tibial plateau (JLOAT) [48]: angle between the proximal tibial line and the horizontal line; **B** Joint line orientation angle by the middle knee joint space (JLOAM) [35]: angle between the middle knee joint space line and the horizontal line; **C** Joint line orientation angle by the femoral condyles (JLOAF) [1]: angle between the distal femoral line and the horizontal line; **D** Medial proximal tibial angle (MPTA) [53]: medial angle between the proximal tibial line and the tibial mechanical axis; **E** Mikulicz joint line angle (MJLA) [53]: medial angle between the middle knee joint space line and the weight-bearing line

As a bony correction technique performed at the proximal tibia, HTO can result in knee joint line obliquity (KJLO) increase, particularly when there is a large correction [1, 4, 35]. Different KJLO measurement methods of joint line orientation angle by the femoral condyles (JLOAF), joint line orientation angle by the middle knee joint space (JLOAM), joint line orientation angle by the tibial plateau (JLOAT), medial proximal tibial angle (MPTA), and Mikulicz joint line angle (MJLA) are described in literature **(**Fig. 1**)** [1, 4, 35, 53].

To the best of our knowledge, there is no published consensus on whether to take a suspected excessive postoperative KJLO into consideration during osteotomy planning. Some studies suggest a double-level osteotomy when there is a predicted excessive postoperative KJLO during HTO planning, which involves a postoperative MPTA > 95° [2, 29, 43] or a postoperative JLOAT > 6° [59]. Another study suggests that HTO is still justifiable despite a predicted slightly excessive postoperative KJLO [14]. A review of current evidence is therefore necessary, with a focus on associations between postoperative KJLO and patient-reported outcome, status of knee ligament and cartilage, radiological outcomes, surgical survival, and outcome of gait analysis or physical function after HTO.

The aim of this paper is to systematically review the literature on the association between KJLO and clinical outcome after HTO for medial knee osteoarthritis and summarize the KJLO cut-off value used when studying this association. We hypothesize that an increase of KJLO after HTO has adverse influences on clinical outcome.

## Methods

This systematic review followed the Preferred Reporting Items for Systematic Reviews and Meta-analyses (PRISMA) guideline [49]. The protocol of this review was preregistered in the PROSPERO registry with no. CRD42022359034.

### Search strategy

A “PEO” method was used to develop the search strategy for this systematic review [42]. The population (P) was defined as patients who underwent HTO for medial knee osteoarthritis. Exposure (E) was defined as postoperative knee joint line obliquity. Outcome (O) was defined as the association between postoperative KJLO and certain clinical outcomes that include the score on a patient-reported outcome measure, assessment of knee cartilage and ligament status, radiological outcome, outcome of gait analysis or physical function, and surgical survival (revision to knee arthroplasty).

Search strategies used in three databases, PubMed, Embase, and Web of Science, are presented in Table 1. Articles were searched from the databases’ inception to 14 September 2022, with an updated search on 9 February 2023 for additional articles. No language restriction was used during the search.

**Table 1:** Search strategy

Database	Search String
PubMed	(“Osteoarthritis, Knee”[Mesh] OR tibia* [tiab] OR knee [tiab]) AND (“Osteotomy”[Mesh] OR osteotom*[tiab]) AND (joint line obliquit* [tiab] OR joint line orientat* [tiab]) AND (outcom* [tiab] OR scor* [tiab] measur* [tiab] OR funct* [tiab] OR test* [tiab] OR exam* OR ligament* [tiab] OR cartilage [tiab] OR musc* [tiab] OR gait [tiab] OR surviv* [tiab] OR fail* [tiab] OR revis* [tiab] OR radiograph* [tiab] OR radilolog* [tiab] OR parameter [tiab])
Embase	(“knee osteoarthritis”/exp OR “tibia*”:ab,ti,kw OR knee:ab,ti,kw) AND (“osteotomy”/exp OR “osteotom*”:ab,ti,kw) AND (“joint line obliquit*”:ab,ti,kw OR “joint line orientat*”:ab,ti,kw) AND (“outcom*”:ab,ti,kw OR “scor*”:ab,ti,kw OR “measur*”:ab,ti,kw OR “funct*”:ab,ti,kw OR “test*”:ab,ti,kw OR “exam*”:ab,ti,kw OR “ligament*”:ab,ti,kw OR “cartilage*”:ab,ti,kw OR “musc*”:ab,ti,kw OR “gait*”:ab,ti,kw OR “surviv*”:ab,ti,kw OR “fail*”:ab,ti,kw OR “revis*”:ab,ti,kw OR “radiograph*”:ab,ti,kw OR “radiolog*”:ab,ti,kw OR “parameter”:ab,ti,kw)
Web of Science	TS = (“knee” OR “tibia*”) AND TS = “osteotom*” AND TS = (“joint line obliquit*” OR “joint line orientat*”) AND TS = (“outcom*” OR “scor*” OR “measur*” OR “funct*” OR “test*” OR “exam*” OR “ligament*” OR “cartilage” OR “musc*” OR “gait” OR “surviv*” OR “fail*” OR “revis*” OR “radiograph*” OR “radiolog*” OR “parameter”)

### Eligibility criteria

Eligible clinical study designs were randomized controlled trials and observational studies including cohort studies, comparative studies, case–control studies and case series (≥ 10 cases). Clinical studies were included in this review when KJLO was measured and the clinical outcome in relation to this KJLO was reported. Nonpatient studies and conference abstracts without full-text were excluded.

### Identification of eligible studies

Endnote software (version 20, Clarivate) was used to exclude duplicates. Based on the predefined eligible criteria, two reviewers (TX and HV) independently screened the studies through three ordered rounds: first titles, then abstracts, and last full-texts. Disagreement between two reviewers was resolved by discussion. If no consensus was achieved, a third reviewer was consulted (IA).

### Data extraction

One reviewer (TX) extracted the following data from included studies: publication year, study location, study design, included knees, mean patient age, mean follow-up time, HTO technique used, KJLO change after HTO, KJLO cut-off value used, and KJLO-related clinical outcome.

### Methodological quality

The modified Downs and Black checklist was used to assess the methodological quality of each included study, with an assessment of study reporting, external and internal validity, and statistical power of patient sample size [9, 61]. Methodological quality was graded by the overall score obtained: excellent (26–28), good (20–25), fair (15–19), and poor (≤ 14) [17, 31]. Two independent reviewers evaluated the methodological quality (TX and HV). Disagreements between the two reviewers were solved by discussion, and a third reviewer was consulted when necessary (IA).

## Results

**Fig. 2:**
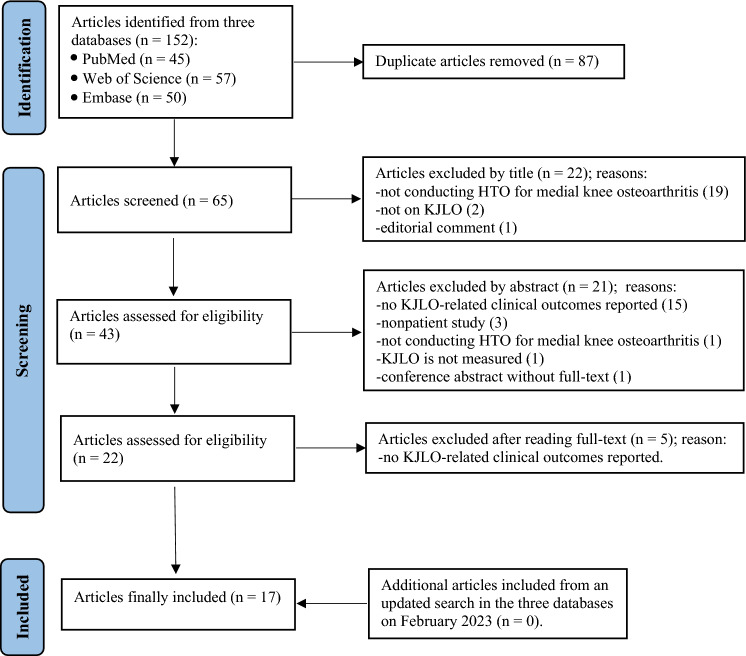
PRISMA flowchart

**Fig. 3:**
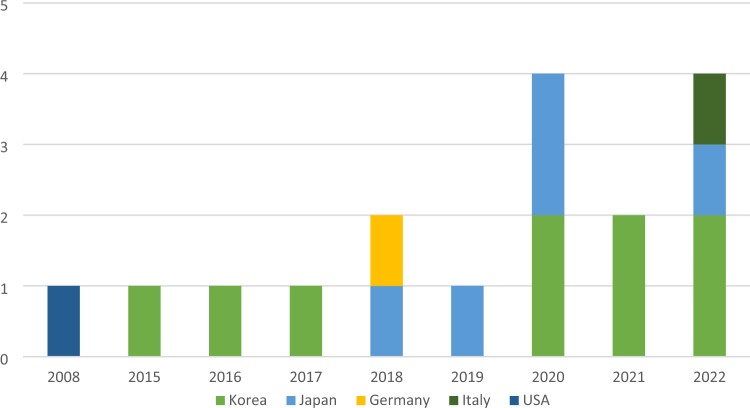
Publication years and study locations

The article selection procedure based on the PRISMA guideline is presented in Fig. 2. A total of seventeen clinical observational studies were included: thirteen cohort studies, three case series, and one case–control study. Fifteen studies performed medial opening wedge HTO, and two studies performed lateral closing wedge HTO. Article publication years and study locations are specified in Fig. 3. The extracted information is depicted in Table 2.

**Table 2:** Summary of findings (17 articles)

Author, year (location)	Study design/Included knees	Patient age/Follow-up time (means, years)	HTO technique (patient groups)	KJLO change after HTO (means)	KJLO cut-off value used and association between KJLO and clinical outcome after HTO
Babis et al. 2008 (USA) [4]	Case series/36	53.0/10.0	LCW HTO	JLOAT (4.17°)	JLOAT postop < 4° is one of the criteria for patients to achieve 10-year surgical survival
Lee KM et al. 2015 (Korea) [35]	Case control/50	53.0/1.0	MOW HTO	JLOAM (4.1°, 0.3°–4.4°)	JLOAM postop is negligibly related to WOMAC and SF-36 (*r* < 0.10)
Oh et al. 2016 (Korea) [48]	Cohort/69	54.4/2.5	MOW HTO (JLOAT postop *-4°–4°,* < -4° or > 4°)	JLOAT (2.1°, -0.7°–1.34°)	Between-group: no significant difference in KSS
Kim CW et al. 2017 (Korea) [26]	Cohort/62	52.3/(> 2.0)	MOW HTO	JLOAM (-0.4°–3.2°)	JLOAM postop > 5° is one risk factor for inferior medial knee cartilage regeneration
Akamatsu et al. 2018 (Japan) [1]	Cohort/86	65.3/2.0	MOW HTO (MPTA postop > *95°*, ≤ 95°)	JLOAF (*5.0°, 0.7°–5.7°*), (3.4°, -0.1°–3.2°) MPTA (*12.5°, 84.7°–97.5°*), (8.5°, 84.6°–93.2°)	MPTA postop > 95°: inferior KSS (range of motion, alignment) and KOOS (sports and recreation) Between-group: no significant difference in KOOS (pain, symptoms, activities of daily living, quality of life), Lysholm score, lateral knee cartilage degeneration, and medial knee cartilage regeneration
Schuster et al. 2018 (Germany) [55]	Cohort/79	50.9/10.0	MOW HTO (MPTA postop > *95*°, ≤ 95°)	MPTA (84.9°–93.2°)	MPTA postop > 95°: inferior IKDC score Between-group: no significant difference in surgical survival rate at 10 years
Goshima et al. 2019 (Japan) [14]	Cohort/94	63.2/6.1	MOW HTO (MPTA postop ≥ *95°*, < 95°)	JLOAT (3.7°, -2.3°–1.4°) MPTA (10.4°, 84.1°–94.5°)	Between-group: no significant difference in JOAS, OKS, KOOS, and lateral knee cartilage degeneration and medial knee cartilage regeneration
Goto N et al. 2020 (Japan) [15]	Cohort/105	61.0/10.2	LCW HTO (MPTA postop ≥ *98°*, ≤ 95°)	MPTA postop (96.6°)	Between-group: no significant difference in KSS
Kim JE et al. 2020 (Korea) [28]	Case series/72	57.1/1.6	MOW HTO	JLOAT (0.79°–2.72°)	JLOAT increase is related to anterior cruciate ligament deterioration (odds ratio 1.6)
Kubota et al. 2020 (Japan) [32]	Cohort/68	60.3/2.5	MOW HTO	JLOAT (1.1°–2.6°)	JLOAT postop is weakly correlated to KOOS (pain, symptom, activities of daily living, sports and recreation, quality of life) (*r* = -0.31, -0.23, -0.30, -0.28, -0.21), physical performance of single-leg standing (*r* = -0.16), isometric muscle strength (quadriceps) (*r* = -0.12), and negligibly correlated with KSS, physical performance of timed up-and-go, isometric muscle strength (hamstrings) (*r* < 0.10)
Song et al. 2020 (Korea) [59]	Cohort/109	58.6/4.6	MOW HTO	Not mentioned	JLOAT postop ≥ 4° is a significant predictor of inferior KSS JLOAT postop ≥ 6° is a significant predictor of inferior KSS and medial joint space narrowing
Kim GW et al. 2021 (Korea) [27]	Cohort/62	(*56.1*, 57.2)/(> 4.0)	MOW HTO (MPTA postop > *95°*, ≤ 95°)	MPTA (*86.8°–96.1°*, 85.8°–90.8°)	MPTA postop > 95°: more patient cases with lateral compartment pain symptom Between-group: no significant difference in WOMAC, KSS, HSSKS, and lateral knee cartilage degeneration
Lee SJ et al. 2021 (Korea) [37]	Cohort/87	59.7/3.0	MOW HTO	JLOAT (2.10°–3.32°)	JLOAT postop is negligibly related to IKDC score (*r* < 0.10)
Kawashima et al. 2022 (Japan) [24]	Case series/39	58.3/2.5	MOW HTO	JLOAT (3.2°, -0.5°–2.8°)	JLOAT increase is weakly related to KOOS (pain) (*r* = -0.12)
Kim JS et al. 2022 (Korea) [29]	Cohort/135	(*57.8*, 55.4, **56.5**, 57.5)/5.6	MOW HTO (MPTA postop *85°– 90°*, 90°– 93°, **93°–95°**, 95°– 102°)	JLOAT (*1.42°*, 1.78°, **1.23°**, 4.08°) MPTA (*5.81°*, 7.31°, **9.05°**, 11.41°)	MPTA postop > 95°: inferior KSS (function) and SF-36 Between-group: no significant difference in WOMAC
Rosso et al. 2022 (Italy) [53]	Cohort/92	53.5/10.8	MOW HTO	MJLA (88.3°–90.6°), MPTA (85.1°–91.5°)	MJLA postop ≥ 94° versus < 94° or MPTA postop ≥ 95° versus < 95°: no significant difference in WOMAC and KSS
Sohn et al. 2022 (Korea) [58]	Cohort/133	56.7/1.0	MOW HTO (MPTA postop > *95°*, ≤ *95°*)	JLOAT (*2.9°, 3.5°–6.0°*), (3.0°, 0.7°–3.7°) MPTA (*10.6°, 85.2°–95.8°*), (8.1°, 83.2°–91.3°)	Between-group: no significant difference in WOMAC and KSS

^a^*postop* postoperative; *KJLO* knee joint line obliquity; *MOW HTO* medial opening wedge high tibial osteotomy; *LCW HTO* lateral closing wedge high tibial osteotomy; *MPTA* medial proximal tibial angle; *MJLA* Mikulicz joint line angle; *KSS* Knee Society Score; *WOMAC* Western Ontario and McMaster Universities Osteoarthritis Index; *KOOS* Knee injury and Osteoarthritis Outcome Score; *IKDC* International Knee Document Committee; *SF-36* Short-Form 36; *HSSKS* Hospital for Special Surgery Knee Score; *JOAS* Japanese Orthopaedic Association Score; *OKS* Oxford Knee Score

^b^For joint line orientation angles (JLOAF, JLOAM, JLOAT), a positive value (+) indicates a medial opening angle, a negative value (-) indicates a lateral opening angle

^c^The design of the included study is determined in accordance with the tutorials of Mathes et al. [40] and Dekkers et al. [8]

^d^The correlation magnitude is graded by the tutorial of Schober et al. [54]

^e^*P* value < 0.05 is considered statistically significant

^f^Italicized or bolded figures correspond to the same groups in a study

### Quality assessment of the included studies

The methodological quality of each included study is presented in Table 3 [1, 4, 14, 15, 24, 26–29, 32, 35, 37, 48, 53, 55, 58, 59]. Three studies were rated as good quality, thirteen as fair quality, and one study as poor quality.

**Table 3:** Methodological quality of included studies by modified Downs and Black checklist

Authors, year	Reporting (top score = 11)	External validity (top score = 3)	Internal validity (bias) (top score = 7)	Internal validity (confounding) (top score = 6)	Power (top score = 1)	Overall score (top score = 28)	Methodological quality grade
Babis et al. 2008 [4]	6	1	4	1	0	12	Poor
Lee KM et al. 2015 [35]	9	2	4	2	1	18	Fair
Oh et al. 2016 [48]	9	2	4	3	1	19	Fair
Kim CW et al. 2017 [26]	8	2	4	3	0	17	Fair
Akamatsu et al. 2018 [1]	10	2	5	4	1	22	Good
Schuster et al. 2018 [55]	8	2	4	2	0	16	Fair
Goshima et al. 2019 [14]	9	2	6	3	1	21	Good
Goto N et al. 2020 [15]	8	1	5	2	0	16	Fair
Kim JE et al. 2020 [28]	8	2	4	3	0	17	Fair
Kubota et al. 2020 [32]	7	2	4	2	0	15	Fair
Song et al. 2020 [59]	9	2	4	2	0	17	Fair
Kim GW et al. 2021 [27]	10	2	6	4	1	23	Good
Lee SJ et al. 2021 [37]	8	2	4	2	0	16	Fair
Kawashima et al. 2022 [24]	8	2	4	3	0	17	Fair
Kim JS et al. 2022 [29]	8	2	5	3	1	19	Fair
Rosso et al. 2022 [53]	10	2	4	3	0	19	Fair
Sohn et al. 2022 [58]	9	2	3	3	0	17	Fair

^a^Methodological quality was graded by the overall score: excellent (26–28), good (20–25), fair (15–19), poor (≤ 14) [17, 31]

### Assessment tools

Patient-reported outcome was assessed by nine different tools in fourteen studies [1, 14, 15, 24, 27, 29, 32, 35, 37, 48, 53, 55, 58, 59] (Table 4). Knee cartilage was assessed arthroscopically in four studies [1, 14, 26, 27] and by medial joint space width (mJSW) in one study [59].

**Table 4:** Tools used for assessing patient-reported outcome

Tools	Used by included studies	Types	Description
Knee Society Score (KSS) [18, 56]	Oh et al. 2016 (old) [48]; Akamatsu et al. 2018 (old) [1]; Goto et al. 2020 (new) [15]; Kubota et al. 2020 (new) [32]; Song et al. 2020 (new) [59]; Kim GW et al. 2021 (old) [27]; Kim JS et al. 2022 (old) [29]; Rosso et al. 2022 (old) [53]; Sohn et al. 2022 (not mentioned) [58]	Knee-specific	Old KSS version: presented in 1989 and updated in 1993 by Dr Insall to assess functional capabilities after knee arthroplasty, including knee score and functional score New KSS version: presented in 2011 by Dr Scuderi, adding objective components including patient treatment expectations, patient satisfaction, and knee activity level to the old version
Western Ontario and McMaster Universities Osteoarthritis Index (WOMAC) [5]	Lee KM et al. 2015 [35]; Kim GW et al. 2021 [27]; Kim JS et al. 2022 [29]; Rosso et al. 2022 [53]; Sohn et al. 2022 [58]	Disease-specific	WOMAC: widely used to assess hip and knee osteoarthritis, including subscales for pain, stiffness, and physical function
Knee injury and Osteoarthritis Outcome Score (KOOS) [52]	Akamatsu et al. 2018 [1]; Goshima et al. 2019 [14]; Kubota et al. 2020 [32]; Kawashima et al. 2022 [24]	Knee-specific	KOOS: used as an extension of WOMAC, adding new items to WOMAC subscales of pain and stiffness as well as adding two new subscales (sports and recreation, quality of life) to assess knee injury
International Knee Document Committee (IKDC) subject knee form [20]	Lee SJ et al. 2021 [37]; Schuster et al. 2018 [55]	Knee-specific	IKDC: developed for outcome measures in patients with knee impairments from ligament/meniscus injury, cartilage lesion and patellofemoral osteoarthritis, including symptoms, sports activity, knee function, and daily living activity assessment
Short-Form 36 (SF-36) [34]	Lee KM et al. 2015 [35]; Kim JS et al. 2022 [29]	Generic	SF-36: a 36-item form widely used to assess health-related quality of life, including both physical and psychological outcome measures
Hospital for Special Surgery Knee Score (HSSKS) [19]	Kim GW et al. 2021 [27]	Knee-specific	HSSKS: designed to assess knee symptoms and function after knee arthroplasty
Japanese Orthopaedic Association Score (JOAS) [3]	Goshima et al. 2019 [14]	Knee-specific	JOAS: used to assess knee function after knee surgery, including pain, function, range of motion, deformity degree, and activities of daily living
Lysholm Score [39]	Akamatsu et al. 2018[1]	Knee-specific	Lysholm score: designed for outcome measures in patients after knee ligament injuries
Oxford Knee Score (OKS) [7]	Goshima et al. 2019 [14]	Knee-specific	OKS: designed for outcome measures in patients after knee arthroplasty with 12 simple questions, including pain, symptoms, and assessment of daily functioning

### Patient-reported outcome

Of the eight included studies assessing the association between postoperative MPTA and postoperative patient-reported outcome, one good-quality study showed inferior Knee injury and Osteoarthritis Outcome Score (KOOS) (sports and recreation) [1], and two fair-quality studies showed inferior Knee Society Score (KSS) (function), Short-Form 36, and International Knee Document Committee (IKDC) scores [29, 55] when postoperative MPTA was > 95°. Two good-quality studies and two fair-quality studies presented no significant differences in KOOS, KSS, Western Ontario and McMaster Universities Osteoarthritis Index score, Japanese Orthopaedic Association Score, Oxford Knee Score, and Hospital for Special Surgery Knee Score between postoperative MPTA > 95° and < 95° [14, 27, 53, 58], and one fair-quality study presented no significant difference in KSS between postoperative MPTA ≥ 98° and ≤ 95° [15].

Out of five fair-quality included studies assessing the association between postoperative JLOAT and postoperative patient-reported outcome, one study showed that postoperative JLOAT ≥ 4° and ≥ 6° were both significant predictors for inferior KSS [59]; another study presented no significant difference in KSS between postoperative JLOAT > 4° and < 4° [48]. A third study stated that postoperative JLOAT was weakly negatively correlated with KOOS and negligibly correlated with KSS [32]; a fourth study showed negligible correlation between postoperative JLOAT and IKDC score [37]. The last of these studies showed weak negative correlation between JLOAT increase post-HTO and postoperative KOOS (pain) [24].

### Knee cartilage

Three good-quality studies showed no significant difference arthroscopically in medial knee cartilage regeneration and lateral knee cartilage degeneration post-HTO between postoperative MPTA > 95° and < 95° [1, 14, 27]. One fair-quality study showed arthroscopically that postoperative JLOAM > 5° was one of the risk factors leading to inferior medial knee cartilage regeneration [26]. Another fair-quality study showed that postoperative JLOAT ≥ 6° was a significant predictor of mJSW narrowing, as assessed by a Rosenberg view X-ray [59].

### Surgical survival

One fair-quality study showed no significant difference in 10-year surgical survival rate between postoperative MPTA > 95° and ≤ 95° [55]. One poor-quality study showed that a postoperative JLOAT < 4° was one of the criteria for achieving 10-year surgical survival after HTO [4].

## Discussion

The most important finding of this review is that there is conflicting evidence on the associations between postoperative KJLO and patient-reported outcome, knee cartilage regeneration, and 10-year surgical survival. Six different KJLO cut-off values are used when studying these associations. Only three of the seventeen included studies meet the criteria of good methodological quality.

The evidence about the association between postoperative KJLO and patient-reported outcome after HTO is conflicting, due to the presence of both supportive and opposite findings on whether a suspected excessive postoperative KJLO is significantly related to an inferior patient-reported outcome. Regarding the supportive findings [1, 29, 55, 59], the patient-reported outcome difference between suspected excessive postoperative KJLO and normal postoperative KJLO also exceeds the published minimal clinically important difference of the assessment tool used [11, 21, 38, 45, 51]. A possible explanation for the current conflicting findings could be that most included studies do not properly match the covariates that can affect postoperative patient-reported outcomes when comparing between suspected excessive postoperative KJLO and normal postoperative KJLO patient groups. This can involve covariates such as patient age, gender, body mass index, preoperative patient-reported outcome, degree of preoperative varus alignment, amount of correction, and postoperative follow-up time [13, 25, 30, 63]. In one study the between-group covariate matching is incorporated into the study design using the propensity score-matching method [27], yet some important covariates such as preoperative patient-reported outcome and amount of correction are not used for propensity score-matching. Some supportive findings should be re-interpreted: Kubota et al. [32] concluded there was a significant correlation between postoperative KJLO and postoperative KOOS (pain, activity daily living, sports and recreation), as the *p* value was < 0.05; however, the correlation coefficient magnitude between postoperative KJLO and the postoperative subscales can be classified as weak, which should be the main outcome rather than whether the correlation is significant or not. Future research should have a better consideration of the covariates that can affect postoperative patient-reported outcome.

The association between postoperative KJLO and medial knee cartilage regeneration after HTO is conflicting, and postoperative KJLO seems not to affect lateral knee cartilage deterioration. A finite element analysis study reported that excessive KJLO (MPTA > 95°) could result in a rapid increase of shear stress in the knee joint [43]. In vitro research shows that abnormal shear stress could induce inflammation and apoptosis of chondrocytes [6, 16, 65], decreasing chondrocyte viability [62]; this may negatively influence cartilage status. However, the above finite element analysis and in vitro findings can only be partially confirmed in clinical research. When comparing between patients with postoperative MPTA > 95° and < 95°, there is no significant difference arthroscopically in medial knee cartilage regeneration and lateral knee cartilage degeneration at mean follow-ups at 1/1.5 years [1, 14, 27]. However, JLOAM > 5° is one of the arthroscopic risk factors for inferior medial knee cartilage regeneration at mean follow-up of 1.9 years, along with the other risk factors which include preoperative severe knee osteoarthritis and a medial knee cartilage bipolar lesion [26]. This conflicting finding may be due to the difference in KJLO measurement method and cut-off value used, as well as the time interval between HTO and follow-up arthroscopy, where a longer time interval benefits medial cartilage regeneration [1, 23]. Also, the difference of lateral knee cartilage degeneration between excessive and normal postoperative KJLO may be evident in a long-term follow-up [1, 14, 27]. Furthermore, a previous study used mJSW on X-ray to assess medial knee cartilage and concluded that JLOAT ≥ 6° was a significant predictor of mJSW narrowing after HTO [59]. However, what the mJSW truly represents remains controversial in recent studies: One study reported that mJSW correlated moderately with knee cartilage thickness on magnetic resonance imaging (MRI) [57], whereas another study reported that mJSW change after HTO reflected the weight-bearing line ratio change on X-ray instead of cartilage regeneration arthroscopically [41]. It is therefore better to use MRI or arthroscopy than mJSW to assess knee cartilage status.

The evidence for the association between suspected excessive postoperative KJLO and long-term surgical survival (revision to knee arthroplasty) after HTO is conflicting. To achieve 10-year surgical survival after HTO, one study stated that patients should have postoperative JLOAT < 4°, postoperative 0–6° valgus alignment, and adequate medial knee loading [4]. Another study found no significant difference in 10-year surgical survival rate between postoperative MPTA > 95° and ≤ 95° patient groups [55]; however, whether between-group covariates were taken into account is not specified. Covariates of patient age, knee cartilage condition, preoperative knee osteoarthritis severity, and postoperative alignment could all affect long-term surgical survival after HTO [10, 22], which may further influence such between-group surgical survival comparisons and the conclusions. Furthermore, although longer operation time has already been described for total knee arthroplasty following HTO than primary arthroplasty [60], an excessive KJLO after HTO might further increase technical challenges when there is a need of conversion to total knee arthroplasty, such as difficulties in restoring soft tissue and ligament balance, joint line height, and mechanics and kinematics of tibiofemoral and patellofemoral joints. In some cases, a stemmed augmented tibial component may be required. Computer assisted three-dimensional planning and simulation may help overcome these difficulties.

There is limited clinical evidence that a KJLO increase after HTO negatively influences the anterior cruciate ligament (ACL), as shown by MRI and arthroscopy in one fair-quality study [28]. Possibly explaining this finding, a previous cadaver study reported that KJLO increase is significantly related to femorotibial subluxation [64]; Ogawa et al. [46, 47] discussed that an abnormal femorotibial subluxation might escalate ligament tension, which might result in ACL deterioration. Not only KJLO increases but also the post-HTO posterior tibial slope increase is found to be related to ACL deterioration [28]. The tibial slope may play a more prominent role than KJLO on ACL status by influencing the ligament strain and laxity in the sagittal plane [12]. Future research could focus on how much KJLO increase is acceptable after HTO.

There is limited clinical evidence that postoperative KJLO is only weakly/negligibly correlated with postoperative physical performance (single-leg standing/timed up-and-go) and isometric muscle strength (quadriceps/hamstrings) after HTO. As discussed by Kubota et al. [32], the two physical performance tests used are too easy for patients to accomplish after HTO, which might be a reason for the weak/negligible correlation determined. A high-demand physical performance test focusing on medial knee loading might result in a better correlation. A previous study reported that postoperative KJLO can affect knee adduction moment after total knee arthroplasty [44], where the knee adduction moment during gait indicates the medial knee contact pressure [33]. Moreover, each HTO-operated patient can present a difference in preoperative KJLO, correction magnitude for targeted alignment, and preoperative physical performance and muscle strength. The influence of KJLO increase after HTO on physical performance test outcomes that determine knee loading should be investigated in future research.

As mentioned in the Introduction concerning the excessive KJLO problem after HTO, double-level osteotomy is suggested when there is a predicted postoperative MPTA > 95° or JLOAT > 6° [2, 29, 43, 59]. Yet again, whether a postoperative MPTA > 95° is associated with inferior clinical outcome after HTO remains uncertain. Also, the proposed 6° JLOAT might not be accurately measured, as the JLOAT measurement can be affected by single-leg and double-leg standing as well as by the bipedal distance used at filming [36, 50]; the patient’s standing position is not well described in the study that proposes a JLOAT of 6° as acceptable KJLO upper limit [59]. According to the present review’s findings, no postoperative KJLO cut-off value is sufficiently supported for clinical usage.

A limitation is that, due to the large variabilities in KJLO measurement methods, KJLO cut-off values, and clinical outcome assessment tools used in the included studies, a meta-analysis could not be performed. Also, there is a lack of the literature regarding the clinical effects of KJLO after double-level osteotomy and varus-producing HTO.

The strength of this systematic review lies in its investigation of the association between postoperative KJLO and clinical outcome, providing a summary of current knowledge for orthopaedic surgeons who perform HTO procedures and are concerned about postoperative KJLO. This review revealed the need of unified KJLO measurement methods and adequate covariate control for future research when assessing the association between postoperative KJLO measurements and clinical outcome.

## Conclusion

Due to the conflicting and limited evidence, the actual association between postoperative KJLO and clinical consequences after HTO for medial knee osteoarthritis cannot be ascertained. The clinical relevance of KJLO after HTO remains controversial.

## Data Availability

The datasets generated during the current study are available from the corresponding author upon reasonable request.

## Abbreviations

ACL: Anterior cruciate ligament
HSSKS: Hospital for Special Surgery Knee Score
HTO: High tibial osteotomy
IKDC: International Knee Document Committee
JLOAF: Joint line orientation angle by the femoral condyles
JLOAM: Joint line orientation angle by the middle knee joint space
JLOAT: Joint line orientation angle by the tibial plateau
JOAS: Japanese Orthopaedic Association Score
KJLO: Knee joint line obliquity
KOOS: Knee injury and Osteoarthritis Outcome Score
KSS: Knee Society Score
MJLA: Mikulicz joint line angle
mJSW: Medial joint space width
MPTA: Medial proximal tibial angle
MRI: Magnetic resonance imaging
OKS: Oxford Knee Score
SF-36: Short-Form 36
WOMAC: Western Ontario and McMaster Universities Osteoarthritis Index
